# 晚期NSCLC维持治疗质疑与问题解析

**DOI:** 10.3779/j.issn.1009-3419.2014.04.02

**Published:** 2014-04-20

**Authors:** 晓晴 刘

**Affiliations:** 100071 北京，军事医学科学院附属307医院肺部肿瘤内科 Department of Lung Cancer, 307 Hospital of PLA, Beijing 100071, China

**Keywords:** 肺肿瘤, 维持治疗, Lung neoplasms, Maintenance therapy

## Abstract

近年来，针对晚期非小细胞肺癌的维持治疗进行了诸多临床研究，而这些研究亦已使得部分药物通过审批应用于临床实践。但是目前维持治疗仍存在诸多不容忽视的问题和质疑，如维持治疗试验设计中存在的缺陷和疏漏；维持治疗评价金标准是什么；获益人群以及维持治疗的药效经济学等问题，本文就上述问题作进一步解析。

## Abstract (en)

Recently, several studies have been carried about maintenance therapy in non-small cell lung cancer, and some chemotherapy agents have been approved for clinical practice. But there are still a lot of questions about maintenance therapy that we have to consider. For example, the defects of design in maintenance therapy trials; how to choose gold standard of maintenance therapy for evaluation; who benefit from the maintenance therapy and how to estimate pharmaceutical economics. In this paper, we will make deep analysis about these issues.

近年晚期非小细胞肺癌（non-small cell lung cancer, NSCLC）治疗新的策略其一即是根据表皮性生长因子受体（epidermal growth factor receptor, *EGFR*）基因突变状态、间变性淋巴瘤激酶（anaplastic lymphoma kinase, *ALK*）融合基因选择一线治疗，对此已达成一致不二的共识。策略其二当属晚期NSCLC维持治疗。细数目前一线化疗，几乎所有药物都进行过维持治疗的尝试，而这些研究亦已使得两种药物（培美曲塞和厄洛替尼）通过注册审批，并写入美国国立综合癌症网络（National Comprehensive Cancer Network, NCCN）指南^[1-10]^（表 1）。而今虽然我们会津津乐道维持治疗的几个阳性试验，并极力推荐维持治疗药物进入临床实践。但闭门思过，我们却不得不思考、直面目前维持治疗存在的数个问题，其始终是我们挥之不去的阴霾，解不开的心结。

**1:** 维持治疗试验 Trails of maintenance chemotherpay

	Study	Agent	PFS (mo)	OS (mo)
Switch maintenance	Fidias^[1]^	Docetaxel Control	5.7^*^ 2.7	12.3 9.7
	JMEN^[2]^	Pemetrexed Control	4.3^*^ 2.6	13.4^*^ 10.6
	SATURN^[3]^	Erlotonib Control	2.8^*^ 2.6	12.0^*^ 11.0
	ATLAS^[5, 6]^	Erlotonib Control	4.8^*^ 3.8	15.9 13.9
	INFORM^[4]^	Gefitinib Control	4.8^*^ 2.6	18.7 16.9
Continuation maintenance	Belani^[7]^	Gemcitabine Control	7.4 7.7	8.0 9.3
	IFCT-GFPC 0502^[8]^	Gemcitabine Erlotonib Control	3.8^*^ 2.9^*^ 1.9	12.1 11.4 10.8
	PARAMOUNT^[9, 10]^	Pemetrexed Control	3.9^*^ 2.6	13.86^*^ 11.1
^*^P < 0.05; PFS: progression free survival; OS: overall survival.				

## 1 无进展生存期（progression free survival, PFS）或总生存期（overall survival, OS），谁才是维持治疗策略评估的金标准？

维持治疗的目的是希望晚期NSCLC患者在完成4个-6个周期一线化疗后疾病稳定以上，身体状况允许时继续治疗，以推迟疾病进展时间，进一步改善患者生活质量，延长总生存。目前大多数维持治疗临床研究的终点采用PFS，由于其出现事件较快，研究时间较短，此外它不受后续治疗的影响。尽管有如此多的优点，但PFS仍然有不准确且存在主观性偏移、易受检查、评估时间影响的缺点。

毫无疑问，从表 1中我们看到，继续治疗（不论是更换新药物还是继续初始方案中非铂部分药物治疗）延长了PFS，但多数研究OS没有获益，或仅仅微弱优势。另外最重要的一点，在这些维持治疗研究中，维持治疗组患者相当于提早接受了二线治疗，而这些药物恰恰在二线治疗研究中与安慰剂对照已被证实是有效的药物，在这种对照组没有接受类似有效治疗的研究设计中，仅仅凭维持组患者PFS获益不能说明这种策略就是正确的。所以OS才应该是维持治疗策略评估明确和决定性的终点。

## 2 提早应用二线药物（转换维持）是否有OS根本获益?

我们知道，根据维持治疗方案的不同，维持治疗临床试验分成两种。第一种，临床研究从标准含铂双药方案转换为不同的单药治疗（转换维持治疗）。第二种，继续给予无铂药物治疗（继续维持治疗）。转换维持治疗实质是关乎二线治疗的最佳时机问题（早治疗/晚治疗）；而继续维持治疗是一线治疗的最佳持续时间问题。

理论上试验组患者（转换维持或早二线治疗）比对照组（晚二线治疗）生存上会更加获益，而且后者必须保证应用了“维持研究药物”。遗憾的是表 1所列举的维持治疗研究中仅Fidias的临床试验设计是早二线治疗与晚二线治疗的直接对照，而结果是试验组患者多西他赛维持与对照组二线多西他赛治疗组OS无差异。其它4项转换维持治疗研究并非完全是早二线对照晚二线研究设计，在对照组进入二线治疗后没能保证全部交叉到维持治疗所用药物的基础上，并有几项研究^[4-6]^OS无差异，所以这一理论假设并没有被这些研究充分证明。

## 3 维持治疗试验中的对照组治疗是否充分？

维持治疗对照组患者疾病进展后是否接受了合适的治疗？这个问题是关乎这些试验成败与否最重要的。表 2显示了有相当部分患者在一线治疗失败后无法接受任何一种治疗，根据既往多项试验结果，这些患者所占比例可高达60%。另外结果（表 2）也显示即使对照组进入二线治疗的患者也不能保证是交叉到维持治疗所用药物。

**2:** 进展时对照组的治疗 Control arm therapy at progression

Study	Agent	Crossover (%)	Anyagent (%)
Fidias^[1]^	Docetaxel	62	62
JMEN^[2]^	Pemetrexed	18	67
SATURN^[3]^	Erlotonib	21	72
ATLAS^[5, 6]^	Erlotonib	40	56
Belani^[7]^	Gemcitabine	N/A^*^	17
IFCT-GFPC 0502^[8]^	Gemcitabine Erlotonib	N/A^*^	91
PARAMOUNT^[9, 10]^	Pemetrexed	N/A^*^	64
INFORM^[4]^	Gefitinib	30 (includes Erlotonib)	67
^*^N/A not applicable, study of continuation maintenance, all patients received drug as part of induction.			

所以在应用某些特定药物进行二线治疗已经明确的时代，这些还未能确保患者已接受了有效二线治疗，就探讨早期治疗还是延迟治疗策略好的研究是不能被简单接受的。简言之，目前维持治疗研究中对照组患者接受了非标准二线治疗，或与维持治疗组患者接受的治疗药物不能够完全平衡匹配，这种偏差势必会影响结果判读的严谨和科学性。

## 4 维持治疗模式确认有效，真正获益者亦并非全部

如果确认一种药物能够使维持治疗组患者PFS、OS都获益，但分层分析结果也显示并非所有接受维持治疗的患者获益均等。那么哪些患者是最能从维持治疗中真正获益呢? SATURN（厄洛替尼转换维持治疗）研究提供了最明确的信息。受益人群是那些疾病稳定（stable disease, SD）的患者，而那些一线治疗获得疾病缓解（完全缓解（complete response, CR）/部分缓解（partial response, PR）的患者完全没有获益。欧洲药品管理局认为，在这一前提下厄洛替尼维持治疗仅限于疾病稳定的患者而不是治疗有效的患者。这一点至关重要。因为这个现象实质上明确了获益的是那些可能已经发生了疾病进展，但按照RECIST（Response Evaluation Criteria in Solid Tumors）标准评价肿瘤增长体积还未达到20%的患者^[11]^。大多数疾病稳定的患者实际上是早期进展的患者。合理的假设是，维持治疗的真正获益者是那些疾病进展缓慢但提早接受了二线治疗的患者。

另一个重要问题，表皮生长因子受体酪氨酸激酶抑制剂（epidermal growth factor receptor tyrosine kinase inhibitor, EGFR-TKI）转换维持治疗是否有益于EGFR野生型患者也是值得关注的。两项临床试验报道的数据（INFORM和SATURN研究）明确显示，酪氨酸激酶活化突变阳性患者的PFS与突变阴性患者不同[INFORM研究的风险比（HR）=0.17，中位PFS为16.6个月*vs* 2.8个月；而SATURN研究的风险比HR=0.10]。第三项临床研究是IIIb期多中心随机安慰剂对照试验，评估了化疗+贝伐珠单抗治疗后，应用贝伐珠单抗+厄洛替尼对比贝伐珠单抗+厄洛替尼安慰剂组治疗局部晚期或转移性NSCLC患者（ATLAS）的安全性和有效性，但该研究结果还没有完全报道。尽管该研究中患者有统计学上的显著获益，但相比突变型患者并不明显。总之，EGFR-TKI维持治疗EGFR患者仅能获得微小的获益。

## 5 维持治疗研究设计不能确保对照组所有患者接受二线治疗

这个问题牵涉到维持治疗研究设计中对照组患者二线治疗介入的时机问题。我们知道，在大多数临床实践中，不参加临床研究的患者在首次发现临床或影像学进展时则有可能就接受二线治疗。而在这个时候，患者的肿瘤大小可能仅增加了10%-15%，而不是RECIST定义的增加20%。恰恰相反，纳入维持治疗临床试验的对照组患者则必须按照RECIST定义，肿瘤增加到20%后才符合二线治疗介入，而此时患者可能疾病已进展到体能状态下降、出现合并症或突然发生死亡，从而丧失了接受二线治疗的机会。这是临床研究设计和规定压制了常规临床实践决策而产生的结果。

我们必须清楚，维持治疗（尤其是转换维持治疗）并不是一种（有效药物）治疗与无任何治疗的对照，而实质是回答早期还是晚期应用有效二线药物的问题。如果不能保证所有对照组患者接受二线有效药物治疗是研究设计方面一个严重漏洞。

## 6 维持治疗研究中4个周期含铂化疗是否已足够？

晚期NSCLC患者推荐进行4个-6个周期含铂化疗，在临床实践中应用6个周期方案治疗十分常见，因为在第4个到第6个周期化疗之间常常能观察到肿瘤的继续缩小。迄今所有维持治疗的临床试验均是对4个周期含铂方案治疗后的患者进行了随机分组，却没有证据显示6个周期治疗后进行维持治疗是有益的。所以我们目前观察到的接受4个周期含铂化疗后继续进行维持治疗PFS获益可能与一线治疗达6个周期的患者获益是一致的。

所以今后有必要设计一项关于维持治疗的随机对照临床试验，一组患者为4个周期化疗后维持治疗，对照组则为6个周期化疗后休息。当然，如有可能6个周期双药化疗后维持治疗组也应考虑，以此设计进行研究观察以回答上述问题。

## 7 继续维持治疗中药物花费与获益

晚期NSCLC一线4个周期含铂化疗结束后，疾病稳定以上患者继续给予无铂药物治疗即继续维持治疗。与换药维持治疗不同，继续维持治疗实质上是提出了一线治疗的最佳持续时间或维持到什么程度最合适这么一个问题。

PARAMOUNT和E4599试验已使得培美曲塞和贝伐珠单抗在化疗结束后继续应用直至病情进展或毒性不能耐受。为了进一步提高与改善维持治疗的效果，近几年开展了多项培美曲塞和贝伐珠单抗联合进行维持治疗的研究^[12-17]^（表 3）。虽然观察终点PFS或OS或多或少改善，结果阳性或阴性，但最让我们临床医生和患者纠结的是，在患者、医疗系统和国家对医疗花费关注逐渐增长的大背景下，越来越豪华的维持治疗药物组合、越来越长的用药时间可能让患者和医疗保险机构不堪重负。而在此种境况下，评价和权衡维持治疗费用和获益比尤为重要！

**3:** 持续维持治疗的临床试验 Trails of continuation maintenance chemotherapy

Study	Agent	PFS (mo) Total/Maintenance population	OS (mo) Total/Maintenance population
E4599^[12]^	Bevacizumab	6.2^*^/N	12.3^*^/17.0
	Control	4.5/N	10.3/16.1
POINTBREAK^[13]^	Bevacizumab+Pemetrexed	6.0^*^/8.6	12.6/17.7
	Bevacizumab	5.6/6.9	13.4/15.7
AVAPERL^[14]^	Bevacizumab+Pemetrexed	7.4^*^/10.2^*^	17.1/19.8
	Bevacizumab	3.7/6.6	13.2/15.9
PRONOUNCE^[15]^	Pemetrexed	4.4/N	10.5/N
	Bevacizumab	5.5/N	11.7/N
ARIES^[16]^	Bevacizumab	N/9.2^*^	N/19.8^*^
	Control (without Bevacizumab)	N/7.9	N/15.3
SAiL^[17]^	Bevacizumab	N/8.9	N/18.8
	Control (without Bevacizumab)	N/4.5	N/8.3
E5508	Bevacizumab	N	N
	Pemetrexed	N	N
	Bevacizumab+Pemetrexed	N	N
^*^P < 0.05; N: not acquire.			

## 8 设计能客观评价维持治疗核心问题的临床试验

鉴于上述提及的目前维持治疗研究中存在的种种问题，我们需要设计合适的研究来正确评价这些方案；首先确定维持治疗获益是否为患者中的某一类人群；另外风险（包括经济风险）和效益都要得到明确评估；在任何治疗开始前就进行随机化，而且必须评估生活质量；一旦非维持治疗组患者出现临床或影像学进展，应接受预先制定的二线治疗，以免对照组由于治疗不足而影响试验客观评价。疾病进展的判定应是常规临床实践决策，而不是必须根据RECIST评价标准。

## 9 结语

几年来维持治疗已走过化疗、小分子靶向、血管靶向、化疗联合靶向之路，失败、成功兼具，失望、希望相伴。加之上述诸如在维持治疗研究中设计缺陷、实施漏洞、结果解读质疑等问题，临床医师必须清醒认识到，虽然药物的适应证已被管理机构批准，但临床实践中不能因此而生搬硬套。维持治疗是一种选择，但不是对所有患者的标准治疗。未来应该在可能获益或适合的亚组中选择患者，设计严谨、科学、合理且独立实施的随机临床研究回答上述质疑和困惑，以最终还晚期NSCLC维持治疗一个真实。
